# Examination of newborn DNA methylation among women with polycystic ovary syndrome/hirsutism

**DOI:** 10.1080/15592294.2023.2282319

**Published:** 2023-11-22

**Authors:** Kristen J. Polinski, Sonia L. Robinson, Diane L. Putnick, Rajeshwari Sundaram, Erin Bell, Paule V. Joseph, James Segars, Weihua Guan, Robert M. Silver, Enrique F. Schisterman, Sunni L. Mumford, Edwina H. Yeung

**Affiliations:** a Division of Population Health Research, Division of Intramural Research, Eunice Kennedy Shriver National Institute of Child Health and Human Development, National Institutes of Health, Bethesda, MD, United States; b Department of Environmental Health Sciences, Epidemiology and Biostatistics, University at Albany School of Public Health, Albany, NY, United States; c Section of Sensory Science and Metabolism, Division of Intramural Research, National Institute on Alcohol Abuse and Alcoholism, National Institutes of Health, Bethesda, MD, United States; d Division of Reproductive Science and Women’s Health Research, Johns Hopkins Department of Gynecology & Obstetrics, Baltimore, MD, United States; e Division of Biostatistics, School of Public Health, University of Minnesota, Minneapolis, MN, United States; f Department of Obstetrics and Gynecology, University of Utah, Salt Lake City, UT, United States; g Department of Biostatistics, Epidemiology and Informatics, Perelman School of Medicine, University of Pennsylvania, Philadelphia, PA, United States

**Keywords:** DNA methylation, polycystic ovary syndrome, testosterone, maternal exposure

## Abstract

Research suggests that polycystic ovary syndrome (PCOS) traits (e.g., hyperandrogenism) may create a suboptimal intrauterine environment and induce epigenetic modifications. Therefore, we assessed the associations of PCOS traits with neonatal DNA methylation (DNAm) using two independent cohorts. DNAm was measured in both cohorts using the Infinium MethylationEPIC array. Multivariable robust linear regression was used to determine associations of maternal PCOS exposure or preconception testosterone with methylation β-values at each CpG probe and corrected for multiple testing by false-discovery rate (FDR). In the birth cohort, 12% (102/849) had a PCOS diagnosis (8.1% PCOS without hirsutism; 3.9% PCOS with hirsutism). Infants exposed to maternal PCOS with hirsutism compared to no PCOS had differential DNAm at cg02372539 [β(SE): −0.080 (0.010); FDR *p* = 0.009], cg08471713 [β(SE):0.077 (0.014); FDR *p* = 0.016] and cg17897916 [β(SE):0.050 (0.009); FDR *p* = 0.009] with adjustment for maternal characteristics including pre-pregnancy BMI. PCOS with hirsutism was also associated with 8 differentially methylated regions (DMRs). PCOS without hirsutism was not associated with individual CpGs. In an independent preconception cohort, total testosterone concentrations were associated with 3 DMRs but not with individual CpGs, though the top quartile of testosterone compared to the lowest was marginally associated with increased DNAm at cg21472377 near an uncharacterized locus (FDR *p* = 0.09). Examination of these probes and DMRs indicate they may be under foetal genetic control. Overall, we found several associations among newborns exposed to PCOS, specifically when hirsutism was reported, and among newborns of women with relatively higher testosterone around conception.

## Introduction

Polycystic ovary syndrome (PCOS) is a heterogeneous condition characterized by androgen excess (hirsutism/hyperandrogenism), menstrual cycle dysfunction, and/or polycystic ovarian morphology and is a common cause of reduced fertility among women of reproductive age [1,2]. PCOS and its related traits cluster in families with an estimated heritability of over 70% [3]. A register-based study of 29,736 daughters found a five-fold increased risk of developing PCOS among daughters of women with PCOS [4]. Though this has been less studied in human populations, both male and female offspring are impacted [5,6]. While daughters are at higher risk of PCOS itself, sons of women with PCOS also have higher risks of obesity and diabetes [7,8]. Genome-wide association studies in PCOS have identified risk alleles involving androgen biosynthesis among others [9]. Yet, these genetic loci explain only about 10% of the heritability [10], indicating that genetic risk alone does not completely predict who will develop PCOS. Evidence from animal models suggests prenatal exposure to excess androgens can have a range of adverse reproductive, metabolic, and behavioural effects on offspring [11], but this has been less studied in human populations [5,6]. This altered intrauterine environment could promote epigenetic modifications that may contribute to a proportion of the remaining risk of developing a PCOS phenotype in the offspring [12,13].

DNA methylation (DNAm) is the most studied epigenetic mechanism in human studies examining associations between intrauterine environmental exposures and offspring outcomes [14]. Findings from animal models revealed differential DNAm in ovarian and adipose tissues after exposure to prenatal androgens [15–17], suggesting that *in utero* exposure to PCOS traits may alter DNAm in offspring. Human studies with small sample sizes have also identified DNAm alterations in multiple tissues, including ovarian tissue [18–21]. Among alterations in the offspring, a small study of 12 participants found that children born to women with PCOS had differential cord blood methylation [18]. In 2–3-month-old infants (*n* = 48), *in utero* PCOS exposure was associated with differences in whole blood DNAm of the promoters in targeted reproductive and metabolic genes (*LEP, LEPR, ADIPOR2, AMH* and *AR*) [19]. These studies highlight the need to generate further evidence in the examination of DNAm in newborns exposed to maternal PCOS and related traits (e.g., androgens) in a larger sample.

Therefore, in a well-characterized cohort, we examined the associations of maternal PCOS and hirsutism diagnoses with newborn dried blood spot DNAm. Additionally, since excess androgen is hypothesized to be a key player in the biological consequences of PCOS, we also explored individual cord blood DNAm differences in offspring of women who did not meet diagnostic criteria for PCOS but had preconception measures of testosterone in a separate cohort.

## Participants, material, and methods

### Study populations

#### Upstate KIDS

The Upstate KIDS Study is a longitudinal cohort study that enrolled 5,034 mothers and 6,171 infants born between 2008 and 2010 in New York State (excluding New York City) [22]. Study recruitment and follow-up procedures have been described elsewhere [22]. Briefly, singleton infants conceived by infertility treatment were identified based on birth certificate data and were frequency matched (1:3) to infants without treatment by region of birth and multiples (e.g., twins, triplets, etc.) [22]. All multiples (twins, triplets, etc.) were invited regardless of infertility treatment status. Parents provided written informed consent for the retrieval of remaining newborn dried blood spots (DBS) from New York State’s Newborn Screening programme when the infants were 8 months old (2009–2011) [23]. Samples were retrieved for the measurement of immunoglobulins, and eluted spots were archived. Parents (of 1071 children) provided additional genetic analysis consent in 2016–2017 to measure DNA methylation from the DBS. For this analysis, a subset of 855 singletons and twins (one randomly selected twin from each pair) with DNA methylation (DNAm) measured were included, as previously described [24]. Follow-up of cohort participants continued until 2019. The New York State Department of Health (IRB #07–097) and the University at Albany (State University of New York; IRB #08–179) institutional review boards approved the study.

#### EAGeR

In an exploratory analysis to evaluate concentrations of hormones, we used data from the Effects of Aspirin in Gestation and Reproduction (EAGeR) trial (2007–2011). EAGeR was a multicenter, double-blind clinical trial that randomized 1,228 women to low-dose aspirin and folic acid vs placebo and folic acid prior to conception [25,26]. The primary objective of the trial was to examine whether preconception-initiated low dose aspirin improved live birth among women with a history of pregnancy loss. Data collected as part of this unique preconception trial have been leveraged into other epidemiologic investigations of the reproductive process. Eligibility for the trial included women aged 18 to 40 who 1) had 1–2 prior pregnancy losses, 2) ≤ 1 prior live birth, 3) ≤ 1 elective termination or ectopic pregnancy, 4) regular menstrual cycles for 21–42 days in the prior year. Exclusion criteria included any prior history of infertility or sub-fertility including conditions like PCOS or currently undergoing or planning use of medical fertility treatments. Women were followed for up to six menstrual cycles while attempting pregnancy and then monthly throughout pregnancy for women who conceived (*n* = 595 delivered a live birth). At the Utah study site – which recruited > 80% of study participants – cord blood was collected beginning in 2009 and was obtained for over 90% of deliveries (*n* = 428). For this analysis, 378 non-Hispanic white singletons with DNAm data were included [27,28]. The study was approved by the IRB (#1002521) at the University of Utah and all participants provided written informed consent prior to enrolling. Supplemental Figure S1 provides a sample size flow chart for both the Upstate KIDS and EAGeR study populations.

### DNA methylation data collection and processing

#### Upstate KIDS

The Upstate KIDS Study’s measurement of DNAm and data processing has been provided elsewhere [24,29]. Briefly, DBS were retrieved from the New York State’s Newborn Screening Program [23]; in which, DNA was extracted from DBS punches [30] using the GenSolve DNA recovery kit (GenTegra, Pleasanton, CA) followed by purification with QIAmp DNA kits (#51104, QIAGEN, Valencia, CA). DNA from the DBS then underwent bisulphite conversion with standardized kits (Zymo EZ DNA Methylation kit; Zymo, Irvine, CA). DNAm was profiled using the Infinium MethylationEPIC 850K BeadChip microarray (Illumina, San Diego, CA) and was processed using the minfi package in R [31]. Low intensity CpG probes with detection p-values >0.01, having > 3% of samples with a bead count < 3, and those missing in > 3% of samples were removed. Quantile normalization and background adjustments of CpG probes were applied, and probes on sex chromosomes were removed as a source of variation. For each cytosine-guanine (CpG) probe, DNAm levels were reported as β-values ranging from 0 (unmethylated) to 1 (methylated).

#### EAGeR

In the EAGeR trial, DNA from cord blood leukocytes was bisulphite converted using the EZ MethylationTM kit (Zymo, Irvine, CA). Illumina’s Infinium MethylationEPIC 850K BeadChip microarray (San Diego, CA) was used to assess DNAm. Data were processed using the minfi package [31] including background and dye-bias corrections and quantile normalization of β-values. Probes on the sex chromosomes were removed. Further details have been published previously [27]. Of note, randomization to low-dose aspirin had no impact on DNAm in cord blood in this preconception cohort [27]. The two cohorts did not measure DNA methylation concurrently in the same batch, and thus data could not be pooled due to batch effects.

### Exposure assessment

In the Upstate KIDS Study, women were mailed questionnaires to capture various exposures including a health history. Women self-reported ever being diagnosed by a doctor or health care provider with PCOS or excessive body hair (i.e., hirsutism) on a questionnaire administered 4 months after delivery. Women were categorized as having PCOS with hirsutism, PCOS without hirsutism, or no PCOS.

Women enrolled in the EAGeR trial attended baseline study visits around day 2–4 of their menstrual cycle prior to randomization. During this preconception visit, women provided blood samples which were processed for plasma and serum. Preconception total testosterone (ng/dL) was analysed by liquid chromatography and tandem mass spectrometry [32,33]. Preconception hormones are more representative of typical hormone concentrations than those during pregnancy [34]. Testosterone was operationalized in two ways for the analysis. First, testosterone was log-transformed and treated as a continuous variable. Second, we categorized women based on quartile of testosterone concentrations using the distribution across the analytic sample with DNAm data available. The top quartile cut-off was > 26.3 ng/dL. Notably, this cut-off is similar to what was observed in the full cohort [33] and is in the range for normal adult women (20–60 ng/dL) [35]. However, previous investigations in this cohort found associations with fecundability even at these levels with reproductive outcomes [33]. Women in the first or lowest quartile (≤15.31 ng/dL) were categorized as the reference category. The periconception period is recognized as a crucial time for the developing epigenome [36], thus having exposure levels prior to pregnancy is uniquely informative.

### Covariates

Covariates for the Upstate KIDS Study were obtained from birth certificate records (parity, plurality, infant sex, maternal age at delivery, pre-pregnancy body mass index [BMI, calculated as weight in kilograms divided by height in metres squared], and use of assisted reproductive technologies) or maternal report at 4 months after delivery (maternal race/ethnicity, maternal educational attainment, and smoking during pregnancy). For EAGeR, pre-pregnancy BMI was obtained from height and weight measured by clinic staff. Additional covariates available included parity, infant sex, and maternal age. Plurality, race/ethnicity, and infertility treatment were not included as the EAGeR sample consisted of singletons, non-Hispanic white race/ethnicity and did not include couples with reported infertility. In both cohorts, other covariates related to DNAm were included. Relative cell count proportions of six leukocyte subtypes and nucleated red blood cells in DBS or cord blood were estimated from a cord blood reference [37]. Plate number was used to adjust for batch effects from the measurement of DNAm microarray. Infant’s DNAm-derived ancestry was inferred using GLINT [38].

### Statistical analysis

Descriptive statistics were tabulated for each study population to compare participant characteristics by exposure status. To examine the array-wide associations of maternal PCOS exposure or testosterone concentrations with methylation β-values at each CpG probe as the outcome, we used multivariable robust linear regression [39]. For maternal PCOS exposure, we conducted two comparisons of newborn DNA methylation levels, one for maternal PCOS with hirsutism compared to no PCOS and one for maternal PCOS without hirsutism compared to no PCOS. Minimally adjusted and fully adjusted models were used. A false discovery rate (FDR) correction based on the Benjamini-Hochberg method was applied to account for multiple testing [40]. Gene annotations within 10Mb were identified using the Illumina database and were verified using the University of California Santa Cruz genome browser (GRCh37/hg19). As a sensitivity analysis, we reran the fully adjusted models excluding 26 newborns in Upstate KIDS with congenital malformations determined through linkage to the New York State Congenital Malformations Registry or maternal report at 4 months after delivery [24]. Sons of women with PCOS also have higher risks of obesity and diabetes [7,8]. Stratification by infant sex was not possible due to sample size among the PCOS with hirsutism exposure group (total *n* = 33, males *n* = 16, female *n* = 17).

In a secondary analysis, we examined differentially methylated regions (DMRs) to determine whether probes in the same epigenetic regions have the same relationship with PCOS exposure, using summary statistics from our array-wide analyses as inputs in the R package dmrff [41]. We defined DMRs as genomic regions which covered a set of CpG probes with nominal EWAS p-values that had at most 1000 base pair (bp) between consecutive probes and were consistently positively or negatively correlated with maternal PCOS with hirsutism. DMRs with Bonferroni threshold of 0.05 were reported. Gene ontology analysis was conducted on the regions identified (at FDR *p* < 0.10) using the missmethyl R package. All analyses were conducted using SAS 9.4 (SAS Institute Inc, Cary, NC) and R 4.0.2 (R Core Team, 2021) [42].

## Results

### Upstate KIDS: self-reported PCOS with or without hirsutism

There were 849 out of 855 infants whose mothers responded to questionnaire items relating to diagnoses of PCOS or excessive body hair. Overall, 12% (102/849) of women had a PCOS diagnosis (8.1% PCOS without hirsutism; 3.9% PCOS with hirsutism). These women were more likely to have a higher pre-pregnancy body mass index, gestational diabetes, or receive fertility treatment to conceive (Table 1).

**Table 1.** Upstate KIDS study maternal characteristics overall and by self-reported PCOS status, *n* = 849.

		PCOS Status		
	Overall(*n* = 849)	No PCOS(*n* = 747)	PCOS without hirsutism(*n* = 69)	PCOS with hirsutism(*n* = 33)
Age, years, mean (SD)	32.0 (5.6)	32.0 (5.7)	31.4 (4.5)	32.8 (4.6)
Education, ≥ some college	587 (69.1)	520 (69.6)	44 (63.8)	23 (69.7)
Race/ethnicity, non-Hispanic White^^^	750 (88.3)	662 (88.6)	59 (85.5)	29 (87.9)
Pre-pregnancy BMI, kg/m^2^, mean (SD)	29.8 (7.0)	26.4 (6.8)	29.0 (7.7)	30.6 (7.5)
Smoking during pregnancy	69 (8.1)	63 (8.4)	6 (8.7)	0 (0.0)
Gestational diabetes	69 (8.1)	52 (7.0)	9 (13.0)	8 (24.2)
Gestational hypertension	72 (8.5)	60 (8.0)	10 (14.5)	2 (6.1)
Fertility treatment	334 (39.3)	254 (34.0)	55 (79.7)	25 (75.8)
Nulliparous	425 (50.8)	371 (50.3)	38 (56.7)	16 (50.0)
Newborns	Overall(*n* = 849)	No PCOS(*n* = 747)	PCOS without hirsutism(*n* = 69)	PCOS with hirsutism(*n* = 33)
Birthweight	3231.5 (688.2)	3225.1 (688.0)	3273.9 (639.1)	3287.8 (799.5)
Gestational age	38.2 (2.4)	38.2 (2.4)	38.3 (1.8)	37.9 (3.0)
Male Sex	452 (53.2)	397 (53.2)	39 (56.5)	16 (48.5)
Singleton	684 (80.6)	601 (80.5)	55 (79.7)	28 (84.9)
C-section	390 (46.1)	347 (46.6)	28 (40.6)	15 (45.5)
Chromosomal Abnormalities	26 (3.06)	24 (3.21)	1 (1.45)	1 (3.03)

^Other race/ethnicity: non-Hispanic Black, 1.6%; Asian, 2.6%; Hispanic, 5.3%; mixed race or other, 2.2%.

Missing data:parity, *n* = 12; c-section, *n* = 3.

Abbreviations: PCOS, polycystic ovary syndrome; SD, standard deviation; BMI, body mass index.

Table 2 provides the FDR-significant CpG probes in the array-wide analysis. Infants exposed to PCOS with hirsutism compared to no PCOS had differential DNAm at cg02372539, cg25209153, cg08471713, cg01185179, and cg17728838. After adjustment for additional covariates including fertility treatment and pre-pregnancy BMI, PCOS with hirsutism remained associated with lower methylation at cg02372539 near the *DACT2* gene [β(SE): −0.081 (0.010); FDR *p* = 0.010], and higher methylation at cg08471713 near the *MEOX1* gene [β(SE):0.077 (0.014); FDR *p* = 0.016] and cg17897916 on chromosome 15 [β(SE):0.050 (0.009); FDR *p* = 0.010]. There was no genomic inflation detected for either the minimally adjusted or fully adjusted models (lambdas of 1.106 and 1.017, respectively). **Supplemental Figures S2 & S3** show the Manhattan and Volcano plots of the results. The methylation levels of cg02372539 and cg08471713 exhibited multimodal distributions, indicating sequence specificity (**Supplemental Figure S4 & S5**). PCOS without hirsutism compared to no PCOS was not associated with individual CpG probes (**Supplemental Table S1**).

**Table 2.** Top probes sorted by FDR p-value for PCOS with hirsutism vs no PCOS, Upstate KIDS.

CpG Probe	β (SE)	FDR p-value	Location	Nearby gene	Relation to
Minimally adjusted model*					
cg02372539	−0.0947 (0.014)	1.55E–05	Chr6: 168703878	*DACT2*	Open Sea
cg25209153	−0.0798 (0.014)	2.05E–03	Chr3: 42093455		Open Sea
cg08471713	0.0844 (0.015)	4.28E–03	Chr17: 41738893	*MEOX1*	Open Sea
cg01185179	0.0087 (0.002)	2.13E–02	Chr17: 7893695		Island
cg17728838	0.0107 (0.002)	3.12E–02	Chr11: 46615784	*AMBRA1*	South Shore
Fully adjusted model^					
cg02372539	−0.0807 (0.010)	0.0095	Chr6: 168703878	*DACT2*	Open Sea
cg08471713	0.0773 (0.014)	0.0164	Chr17: 41738893	*MEOX1*	Open Sea
cg17897916	0.0501 (0.009)	0.0095	Chr15: 62681461		North Shore

*Minimally adjusted model adjusted for infant sex, plurality, cell type, and batch.

^^^Fully adjusted model adjusted for infant sex, plurality, cell type, batch, epigenetically derived ancestry, maternal race, maternal educational attainment, maternal age, maternal prepregnancy BMI, parity, and use of assisted reproductive technologies.

Abbreviations: PCOS, polycystic ovary syndrome; SE, standard error; FDR, false discovery rate.

Results were similar in the sensitivity analysis when we removed 26 newborns with congenital anomalies of potential genetic aetiology (*n* = 2 with intrauterine exposure to PCOS) (**Supplemental Table S2**). Both cg08471713 and cg17897916 remained significantly associated with PCOS with hirsutism. Further, infants exposed to PCOS without hirsutism had lower methylation at cg04578890 [β(SE): −0.048 (0.009); FDR *p* = 0.036]. This CpG was previously identified as a top-ranked probe in the analysis conducted in the full sample prior to excluding congenital anomalies as shown in **Supplemental** Table S1.

In the regional analysis, we identified 8 differentially methylated regions (DMRs) using a 1000bp window in the association of self-reported PCOS with hirsutism (Table 3). **Supplemental Figures S6 & S7** show the Manhattan and Volcano plots of the results. These 8 DMRs corresponded to nearby genes: *TMEM237, WDR53/FBXO45*, *NAA15, TRIM26, BRD2, RASAL1, PAGR1/C16orf53 and GATA5*. CpGs within these DMRs did not overlap with or were close to the FDR-significant probes identified in the individual CpG models. Gene ontology analysis using missMethyl yielded no significant pathways.

**Table 3.** Differentially methylated regions (DMR) identified in PCOS with hirsutism vs. no PCOS analysis, Upstate KIDS.

Chr	Positions	# of CpG probes*	Nearby Gene(s)	Function^†^	β (SE)	Bonferroni p-value
2	202506468- 202507561	4	*TMEM237*	Required for ciliogenesis; enhanced expression in retina	0.363 (0.07)	0.027
3	196295364- 196296266	3	*WDR53/FBXO45*	Unknown function (WDR53); Tissue enriched (testis)/Role in neurodevelopment; low tissue specificity (FBXO45)	−0.523 (0.10)	0.011
4	140222502–140223687	6	*NAA15*	Vascular and neuronal growth and development; associated with neurodevelopmental delays; low tissue specificity	0.421 (0.07)	0.001
6	30181425–30181742	3	*TRIM26*	Associated with neural tube defects; low tissue specificity	0.542 (0.10)	0.012
6	32937355 – 32940489	11	*BRD2*	Possible role in spermatogenesis or folliculogenesis; low tissue specificity	0.372 (0.06)	<0.001
12	113573421–113573809	4	*RASAL1*	Involved in cellular proliferation and differentiation; enhanced expression in the parathyroid	0.436 (0.08)	0.01
16	29826926–29827345	3	*PAGR1/C16orf53*	Important for cell survival; enhanced expression in the ovary	0.496 (0.10)	0.040
20	61050885- 61051795	5	*GATA5*	Cardiovascular development; enhanced expression in intestine, testis, urinary bladder	0.346 (0.10)	0.02

*Defined as genomic regions covering a set of nominal EWAS p-value CpG probes with at most 1000bp between consecutive probes that were consistently positively or negatively correlated with the exposure.

^†^Look up in the Human Protein Atlas (proteinatlas.org).

Abbreviations: PCOS, polycystic ovary syndrome; SE, standard error.

### Exploratory analysis: preconception testosterone

The Effects of Aspirin in Gestation and Reproduction (EAGeR) analytic sample consisted of 351 (90%) out of 391 non-Hispanic white participants with testosterone data. Median (min, max) testosterone was 20.40 (5.27, 73.99) ng/dL (Table 4). Higher preconception total testosterone tended to be found in women with lower maternal age, higher pre-pregnancy BMI or being nulliparous.

**Table 4.** EAGeR maternal characteristics overall and by testosterone quartiles, *n* = 351.

		Testosterone (min-max)				
	Overall	Quartile 1 (5.27–15.31 ng/dL)	Quartile 2 (15.34–20.37 ng/dL)	Quartile 3 (20.4–26.31 ng/dL)	Quartile 4 (26.35–73.99 ng/dL)	p-value*
Testosterone ng/dL, median (IQR)	20.4 (11.6)	12.7 (2.7)	18.0 (2.2)	22.6 (3.1)	30.9 (9.2)	
Age, years, mean (SD)	28.3 (4.4)	29.3 (4.6)	29.0 (4.4)	27.7 (4.2)	27.4 (4.2)	0.0083
Education, > high school	317 (90.3)	81 (90.0)	80 (94.1)	75 (89.3)	81 (88.0)	0.8810
Pre-pregnancy BMI, kg/m^2^, mean (SD)	25.1 (5.4)	24.4 (5.7)	24.1 (4.8)	25.7 (5.1)	26.1 (5.7)	0.0359
Smoker, never	326 (92.9)	86 (95.6)	78 (91.8)	78 (92.9)	84 (91.3)	0.0797
Parous, 1–2 prior live births	212 (60.4)	62 (68.9)	53 (62.3)	54 (64.3)	43 (46.7)	0.0144
Age at menarche, years, mean (SD)	12.8 (1.4)	12.8 (1.5)	12.9 (1.5)	12.6 (1.3)	12.8 (1.4)	0.4662
Number of periods 6 months prior to baseline, mean (SD)	4.3 (1.6)	4.3 (1.6)	4.5 (1.5)	4.3 (1.7)	4.3 (1.4)	0.8775
Menstrual regularity 12 months prior to baseline, yes	288 (84.0)	75 (84.3)	70 (83.3)	71 (88.7)	72 (80.0)	0.1471

All values n (%) unless otherwise stated.

* ANOVA p-values presented for continuous characteristics and chi-square or Fisher’s exact p-values for categorical characteristics. Missing data: BMI, *n* = 3; age at menarche, *n* = 12; number of periods, *n* = 13; menstrual regularity, *n* = 8. Abbreviations: EAGeR, Effects of Aspirin in Gestation and Reproduction; SD, standard deviation; BMI, body mass index.

The top FDR p-value ranked CpGs from the array-wide analysis are presented in Table 5. We did not find FDR-significant associations with DNAm when we examined testosterone as a continuous variable. Exposure to the top quartile of testosterone compared to the lowest quartile was marginally associated with increased newborn DNAm at cg21472377 [β(SE): 0.019 (0.004); FDR *p* = 0.0911] (**Supplemental Table S3**). All models were adjusted for infant sex, maternal age, cell type, batch, maternal pre-pregnancy BMI and parity.

**Table 5.** Top probes sorted by FDR p-value for testosterone analysis, EAGeR*.

CpG Probe	β (SE)	FDR p-value	Location	Annotation	Relation to Island
Testosterone (log), ng/dL					
cg24233293	−0.0032 (0.001)	0.1602	Chr10:130303915		Open Sea
cg04152675	0.0009 (0.0002)	0.1602	Chr2:202085364	*CASP10*	Open Sea
cg00280270	0.0004 (0.0001)	0.1884	Chr22:21320131	*AIFM3*	South Shore
cg22628339	−0.0012 (0.0003)	0.4031	Chr7:134296773		Open Sea

*Adjusted for infant sex, maternal age, cell type, batch, maternal pre-pregnancy BMI and parity.

Abbreviations: EAGeR, Effects of Aspirin in Gestation and Reproduction; SE, standard error; FDR, false discovery rate.

In the regional analysis, we identified 3 DMRs using a 1000bp window between consecutive probes in association with testosterone (continuous, log transformed) (**Supplemental Table S3**). These DMRs corresponded to nearby genes: *EHMT2, TNFAIP8* and *TOLLIP*. CpGs within these DMRs did not overlap with DMRs identified to be associated with PCOS with hirsutism in the Upstate KIDS cohort.

## Discussion

We found several differences in newborn methylation at individual CpG probes by maternal PCOS with reported hirsutism, though the difference in methylation near the *DACT2* and *MEOX1* genes may be due to downstream genetic control. Of interest were 8 DMRs identified, each comprised of multiple consecutive CpG probes located within a 1000 base pair window in genes with range of function. We found limited differences in newborn methylation at individual CpG probes by maternal PCOS without reported hirsutism. In the exploratory analysis of testosterone in an independent preconception cohort of women without PCOS, we found no differences in newborn methylation at individual CpG probes when testosterone was modelled as a continuous variable, although we identified 3 DMRs. However, the top quartile of circulating testosterone (≥26.35) was marginally associated with 1 CpG probe near a long non-protein coding gene on chromosome 6.

We showed that infants exposed to PCOS with hirsutism had decreased DNAm at cg02372539 near the *DACT2* gene on chromosome 6, as well as increased DNAm at cg08471713 near the *MEOX1* gene on chromosome and cg17897916 on chromosome 15 after adjustment for potential confounders, including pre-pregnancy BMI. *DACT2* (or *DAPPER2*) is a key factor in Wnt signalling involved in the regulation of embryonic development [43]. *MEOX1* functions in somite development and haemopoietic stem cell differentiation [44]. Further, *MEOX1* is responsible for regulating fibroblast plasticity and its expression may be upregulated in cardiac fibrosis [45]. According to a publicly available database [46], nearby methylation quantitative trait loci (*cis*-mQTL) are associated with DNAm at cg08471713 at birth. This indicates that DNAm at this CpG probe may be partially under foetal genetic control which was further supported by the multimodal distribution of methylation levels at this probe. Examination of CpG probes identified in the DMR analysis also identified mQTLs at birth near *NAA15*, *TMEM237, GATA5* and *RASAL1*, suggesting genetic determinants of DNAm at these probes from the DMR. Of note, these individual CpG probes and regions were not near loci identified in a previous genome-wide meta-analysis of PCOS [9]. Given that previous research characterizes PCOS as a phenotype determined by multiple genes and environmental factors [13], it is not surprising that we are seeing possible variation in multiple loci that may influence DNAm. These DMRs were also located in genes with a range of function reflecting the wide-ranging consequences that have been previously identified with PCOS and hirsutism, including reproductive, neurobehavioral, and cardiometabolic [47–51].

We found a single difference at cg04578890 based on in-utero exposure to maternal PCOS without hirsutism when we removed 26 newborns with congenital anomalies. This probe is located on chromosome 20 and mapped to two nearby genes: *FKBP1A-SDCBP2*, with an unclear functional role in humans, and *SDCBP2-AS1*, which has been linked to ovarian cancer [52]. The lack of differences among infants exposed to PCOS without hirsutism may be attributed to the heterogeneity of the syndrome. One potential explanation is that women who reported PCOS with hirsutism had higher circulating androgens, as levels are known to be higher in pregnant women with PCOS confirmed by the Rotterdam criteria [53,54]. As we were unable to confirm preconception androgen levels within the Upstate KIDS Study, we leveraged an independent preconception cohort of women. However, the top CpG probes identified in the testosterone analysis did not overlap with those identified in the PCOS with hirsutism analysis. The examination of testosterone levels of women without PCOS presents an examination of associations not complicated by downstream treatments, but if a threshold level of testosterone is necessary to see the same associations, it could explain the differences in results. Another explanation may be related to insulin resistance [50,55] as current recommendations from the American College of Obstetricians and Gynecologists (ACOG) include improving insulin sensitivity prior to pregnancy via treatments like metformin, which in turn can decrease circulating androgens [56]. Additional PCOS phenotyping is needed to disentangle the underlying reasons.

A unique aspect of this study was the ability to examine DNAm differences in neonates of women without PCOS who had preconception serum measures of testosterone in an independent cohort. Periconception is an important time for embryonic and placental development, and exposures during this time are important in the developing epigenome [57]. Preconception testosterone is more representative of typical testosterone concentrations than those measured during pregnancy [34]. Exposure to the top quartile of preconception testosterone (>26.3 ng/dL) was marginally associated with increased DNAm at cg21472377 near an uncharacterized long noncoding *LOC100505530* locus. No other differences in newborn methylation were found when testosterone was modelled as a continuous variable. It’s possible that preconception testosterone may be affecting epigenetic alterations in oocyte DNA rather than in the zygote after fertilization, which may explain the lack of associations seen between preconception testosterone and cord blood DNAm. Notably, average testosterone levels of 22.16 ng/dL in the preconception cohort are within the normal range for adult women [35]; however, other research has found increases in anovulatory cycles with ‘higher’ testosterone within a normal range [58]. Further analysis is needed to examine how these associations compare in women with testosterone outside of the normal range like those seen in women with PCOS [53]. Further examination of these hormone levels over the course of pregnancy may have a more direct effect on cord blood DNAm.

This study relied on DNAm from DBS and cord blood samples. Previous studies have compared DNA methylation levels from these sources [59,60]. In terms of the impact of handling the material, one group tested differences by putting the same cord blood sample on a DBS card stored at room temperature for 7 days compared to the blood immediately frozen, finding minimal differences by these storage conditions [59]. Unlike DBS, cord blood may be contaminated by maternal blood [61]. Another study used paired samples of cord blood and DBS collected from the same infants and found high agreement for the majority of CpGs (70.1%) [60]. Other CpG sites varied in correlations, but they acknowledged that it has been noted that white blood cell type proportions differ between these two sources of blood and therefore it should not be surprising that the DNAm levels, not accounting for cell type, would differ. Nevertheless, the study authors recommended that comparison of mean levels in such situations is appropriate. We have conducted study specific analyses and furthermore adjusted for cell type.

Strengths of this study include two well-characterized cohorts with numerous covariates available and the examination of newborn DNAm in the context of preconception exposure to total testosterone in EAGeR. Limitations of this study include the inability to evaluate other tissues like adipose tissue [62], a smaller sample size of women with PCOS which can limit power, though the proportion is similar to national estimates, and assessing PCOS exposure using maternal report of a physician diagnosis rather than diagnostic criteria (e.g., NIH [63], Rotterdam [54]). However, in epidemiologic studies, like Upstate KIDS, it is not feasible to clinically verify PCOS status and in clinical practice information such as hair patterning is essentially based on women’s own report (given women will shave, etc) and self-reported PCOS has been viewed as a valid alternative approach, including studies investigating its genetic aetiology [64,65]. While preconception testosterone is more typical of T concentrations than those measured throughout pregnancy, we cannot account for diurnal rhythms due to availability of a single measure which was not meant for diagnostic purposes. Lastly, stratification by subgroups (e.g., infant sex, fertility treatment, etc.) could not be conducted due to the small number of women reporting PCOS with hirsutism. However, none of the current CpG sites identified were associated with use of fertility treatment in a previous investigation [24].

## Conclusion

Overall, our findings suggest that excess circulating maternal androgens may potentially alter DNAm of offspring in infancy, but several CpG probes and DMRs identified in both the individual and regional analysis may be under foetal genetic control based on nearby mQTLs. We also found limited evidence of individual DNAm differences in offspring of women who did not meet the diagnosis of PCOS but had preconception measures of testosterone in the upper quartile of the preconception cohort (>26.3 ng/dL). This exploratory study should be replicated, and further research in independent cohorts is needed. Pregnant women with PCOS should continue to work with their health care provider to promote a healthy pregnancy and safe delivery.

## Supplementary Material

Supplemental PCOS_DNAm_EPIGENETICS TRACK-copy.docx

KEPI_2023_0106R1 Response 10302023.docx

## Data Availability

Data that support the findings of this study are available on request from the corresponding author [EY]. Data are not on a public database due to New York State restrictions (i.e., releasing information that could compromise participant privacy/consent).

## Clinical trial registration number (EAGeR):

#NCT00467363
