# Supplementary material for: Examination of newborn DNA methylation among women with polycystic ovary syndrome/hirsutism
Source: Epigenetics. 2023 Nov 22;18(1):2282319. doi: 10.1080/15592294.2023.2282319 (PMC10732621; doi:10.1080/15592294.2023.2282319)
Supplement: Supplemental PCOS_DNAm_EPIGENETICS TRACK-copy.docx [file KEPI_A_2282319_SM2010.docx]

**Supplemental Tables**

**Examination of newborn DNA methylation among women with polycystic ovary syndrome/hirsutism**

Kristen J. Polinski^1^, Sonia L. Robinson^1^, Diane L. Putnick^1^, Rajeshwari Sundaram^1^, Erin Bell^2^, Paule V. Joseph^3^, James Segars^4^, Weihua Guan^5^, Robert M. Silver^6^, Enrique F. Schisterman^7^, Sunni L. Mumford^7^, Edwina H. Yeung^1^

**Supplemental Table 1.** Top probes ordered by FDR p-value for PCOS without hirsutism vs. no PCOS comparison, Upstate KIDS

|  | PCOS w/out hirsutism | | | PCOS with hirsutism | | |  |  |  |
| --- | --- | --- | --- | --- | --- | --- | --- | --- | --- |
| CpG Probe | β | SE | FDR p-value | β | SE | FDR p-value | Location | Annotation | Relation to Island |
| Minimally adjusted model* | | | | | | | | | |
| cg26420566 | 0.0218 | 0.005 | 0.5537 | -0.0044 | 0.007 | 0.9606 | Chr6:8086774 | *SCARNA27; EEF1E1* | Open Sea |
| cg24991452 | 0.0023 | 0.001 | 0.5537 | 0.0005 | 0.001 | 0.9639 | Chr6:35996059 | *MAPK14* | Island |
| cg06260964 | -0.0272 | 0.006 | 0.5537 | 0.0027 | 0.009 | 0.9846 | Chr7:2473003 | *CHST12* | Island |
| cg00075192 | -0.0071 | 0.002 | 0.5537 | 0.0034 | 0.002 | 0.8593 | Chr7:42951814 | *C7orf25* | Island |
| cg08913010 | -0.0211 | 0.005 | 0.5537 | -0.0025 | 0.007 | 0.9812 | Chr11:7272653 | *SYT9* | North Shore |
| cg22436280 | -0.0315 | 0.007 | 0.5537 | 0.0173 | 0.010 | 0.8151 | Chr16:77709505 |  | Open Sea |
| cg22257099 | 0.0159 | 0.003 | 0.5537 | -0.0073 | 0.005 | 0.8503 | Chr17:17139986 | *FLCN* | North Shore |
| cg09413013 | 0.0274 | 0.006 | 0.5537 | -0.0185 | 0.009 | 0.7601 | Chr17:76137024 | *TMC8* | Island |
| cg04578890 | -0.0397 | 0.008 | 0.5537 | -0.0038 | 0.012 | 0.9848 | Chr20:1349052 | *FKBP1A-SDCBP2; SDCBP2-AS1* | Open Sea |
| Fully adjusted model^^^ | | | | | | | | | |
| cg00075192 | -0.0075 | 0.002 | 0.4058 | 0.0019 | 0.002 | 0.9904 | Chr7: 42951814 | *C7orf25* | Island |
| cg22257099 | 0.0157 | 0.003 | 0.4058 | -0.0082 | 0.005 | 0.9695 | Chr17: 17139986 | *FLCN* | North Shore |
| cg21736309 | -0.0076 | 0.002 | 0.4058 | 0.0001 | 0.002 | 0.9995 | Chr19: 19774664 | *ATP13A1* | Island |
| cg04578890 | -0.0438 | 0.009 | 0.4058 | -0.0037 | 0.012 | 0.9979 | Chr20:1349052 | *FKBP1A-SDCBP2; SDCBP2-AS1* | Open Sea |

*Model adjusted for infant sex, plurality, cell type, and batch

^^^ Model adjusted for infant sex, plurality, cell type, batch, epigenetically derived ancestry PCs, maternal race, maternal educational attainment, maternal age, maternal prepregnancy BMI, parity, and use of assisted reproductive technologies.

Corresponding β coefficients and FDR p-values from the PCOS with hirsutism vs. no PCOS comparison in the model are also provided for comparison.

Abbreviations: PCOS, polycystic ovary syndrome; SE, standard error; FDR, false discovery rate.

**Supplemental Table 2.** Sensitivity analysis of PCOS associations removing congenital malformations*, Upstate KIDS

|  | **PCOS with hirsutism vs. no PCOS** | |  | | |
| --- | --- | --- | --- | --- | --- |
| **CpG Probe** | **β (SE)** | **FDR p-value** | **Location** | **Nearby gene** | **Relation to** |
| cg08471713 | 0.0813 (0.014) | **0.0051** | Chr17:41738893 | *MEOX1* | Open Sea |
| cg17897916 | 0.0490 (0.009) | **0.0264** | Chr15:62681461 |  | North Shore |
| cg04578890 | -0.0479 (0.009) | **0.0360** | Chr20:1349052 | *FKBP1A-SDCBP2;*  *SDCBP2-AS1* | Open Sea |

Adjusted for infant sex, plurality, cell type, batch, epigenetically derived ancestry PCs, maternal race, maternal educational attainment, maternal age, maternal prepregnancy BMI, parity, and use of assisted reproductive technologies.

*hereditary anemia, trisomy 21, cerebral palsy, limb deficiency, hypospadias, hemophilia, supernumerary digit, hemolytic RH disease, congenital cyst, double right kidney, congenital hypoplastic left heart, craniosynostosis, chondrodystrophy, factor VIII, congenital hydrocephalus, omphalocele, Edwards Syndrome, cleft lip/palate.

Abbreviations: PCOS, polycystic ovary syndrome; SE, standard error; FDR, false discovery rate.

**Supplemental Table 3.** Top probes sorted by FDR p-value for testosterone (quartile) analysis, EAGeR*

|  | Q4 vs Q1 | | Q3 vs Q1 | | Q2 vs Q1 | |  |  |  |
| --- | --- | --- | --- | --- | --- | --- | --- | --- | --- |
| CpG Probe | β (SE) | FDR p-value | β (SE) | FDR p-value | β (SE) | FDR p-value | Location | Annotation | Relation to Island |
| cg21472377 | 0.0189 (0.004) | 0.0911 | 0.0095 (0.004) | 0.8382 | 0.0113 (0.004) | 0.9999 | Chr6:37474946 | *LOC100505530* | Open Sea |
| cg09758180 | 0.0228 (0.005) | 0.2033 | 0.0154 (0.005) | 0.7122 | 0.0132 (0.005) | 0.9999 | Chr12:109839021 | *MYO1H* | Open Sea |
| cg19160672 | 0.0116 (0.002) | 0.2184 | 0.0050 (0.002) | 0.8947 | 0.0030 (0.002) | 0.9999 | Chr6:90635241 |  | Open Sea |
| cg09810483 | 0.0103 (0.002) | 0.2184 | 0.0021 (0.002) | 0.9734 | 0.0069 (0.002) | 0.9999 | Chr12:122624633 | *MLXIP* | Open Sea |

*Adjusted for infant sex, maternal age, cell type, batch, maternal pre-pregnancy BMI and parity

Abbreviations: EAGeR, Effects of Aspirin in Gestation and Reproduction; SE, standard error; FDR, false discovery rate

**Supplemental Table 3.** Differentially methylated regions (DMR) identified in preconception total testosterone analysis, EAGeR

| # of CpG probes | Chromosome | Positions | Nearby Gene(s) | Function^†^ | β (SE) | Bonferroni p-value |
| --- | --- | --- | --- | --- | --- | --- |
| 4 | 6 | 31852322 - 31853518 | *EHMT2* | Encodes a methyltransferase that methylates histone (H3) at lysine 9; low tissue specificity | -0.134 (0.03) | 0.0375 |
| 2^^^ | 5 | 118691406 - 118691751 | *TNFAIP8* | Functions in the suppressing TNF-mediated apoptosis; enhanced expression in lymphoid tissue, enhanced in granulocytes | 0.482 (0.10) | 0.0415 |
| 2^^^ | 11 | 1304396 - 1304875 | *TOLLIP* | Role in regulated inflammatory signaling; enhanced in spermatids | 0.202 (0.04) | 0.0487 |

*Defined as genomic regions covering a set of nominal EWAS p-value CpG probes with at most 1000bp between consecutive probes that were consistently positively or negatively correlated with the exposure.

^†^Look up in the Human Protein Atlas (proteinatlas.org) (1)

Abbreviations: PCOS, polycystic ovary syndrome; SE, standard error.

**Supplemental Figures**

**Supplemental Figure 1.** Study Flowchart, Upstate KIDS(2) & EAGeR(3)

**Upstate KIDS EAGeR**

428 with cord blood

4989 mothers^a^ (enrolled

2008-2010)

3107 mothers^b^

(DBS consent)

2009-2011)

849 newborns (n=69 with mothers who had PCOS without hirsutism; 33 with mothers who had PCOS with hirsutism; 747 with mothers without PCOS)

351 newborns with DNA methylation and paired testosterone data

391 newborns with DNA methylation data passing QC

398 with

sufficient DNA

855 unrelated^c^ newborns (with DNA methylation)

913 mothers (of 1071 newborns with genetic consent

2016-2017)

**Supplemental Figure 2.** Manhattan plot corresponding to Table 2 results of PCOS-hirsutism EWAS (fully adjusted model)


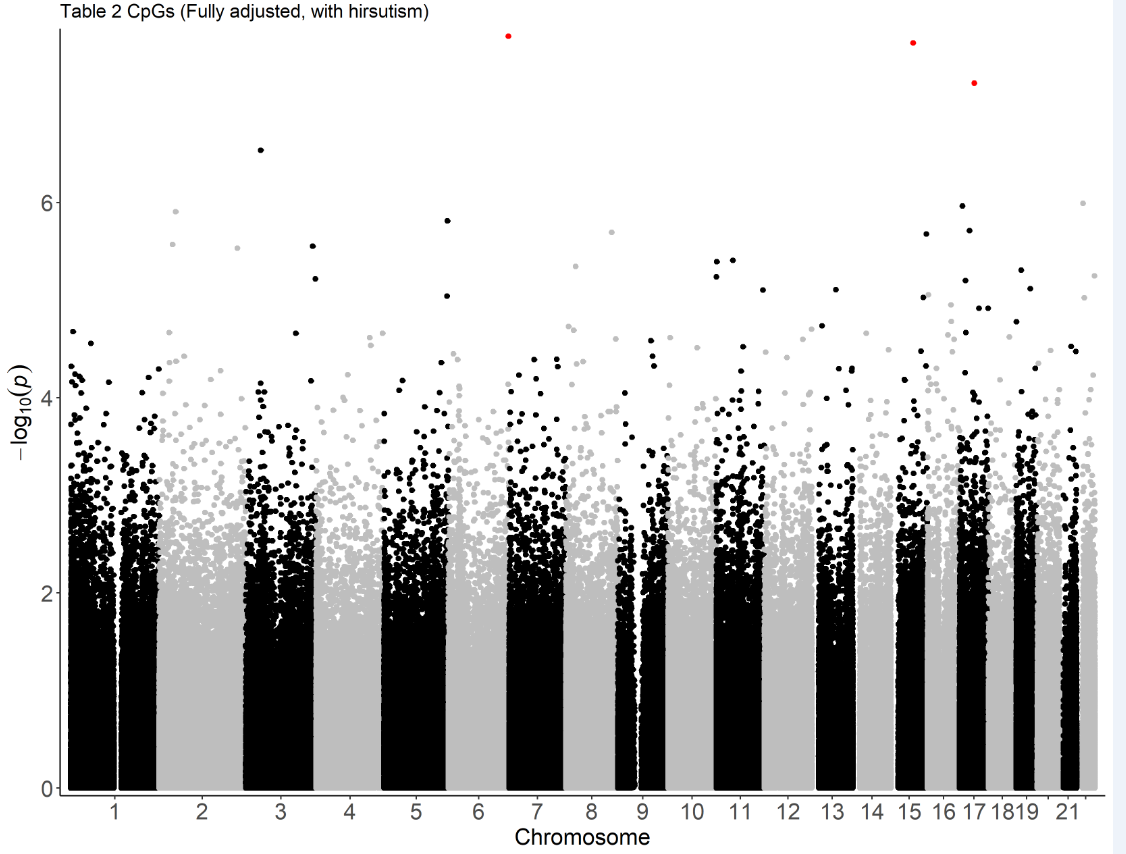


Legend: Colored dots denote the three FDR-significant CpGs identified in offspring newborn blood samples with exposure to maternal PCOS with hirsuitism compared to no PCOS on chromosomes 6, 15, and 17, respectively.

**Supplemental Figure 3.** Volcano plot corresponding to Table 2 results of PCOS-hirsutism EWAS (fully adjusted model)


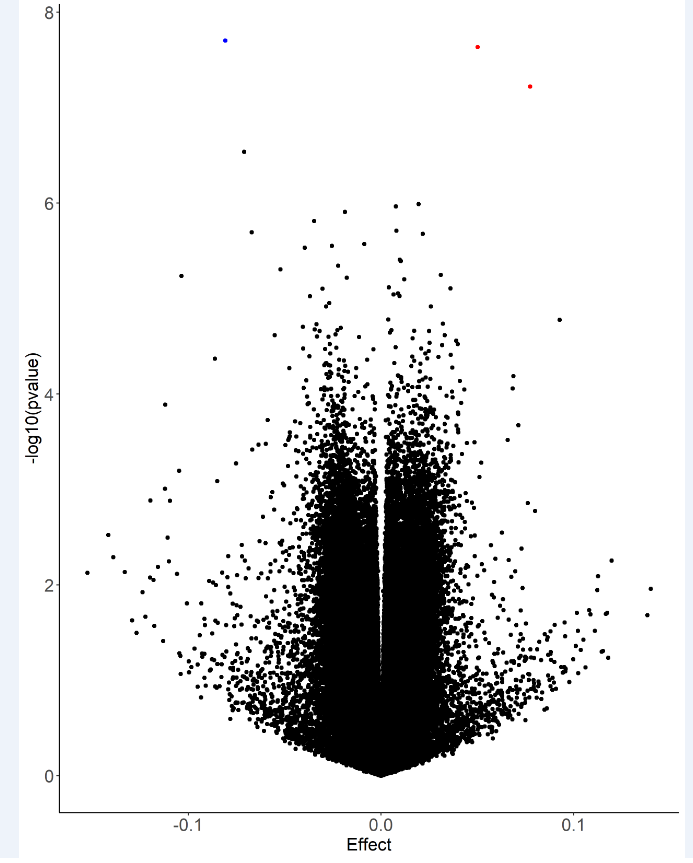


Legend: Colored dots denote the three FDR-significant CpGs identified in offspring newborn blood samples with exposure to maternal PCOS with hirsuitism compared to no PCOS. The blue denotes less methylation with exposure (at cg02372539) and the red denotes more methylation with exposure (at cg08471713 and cg17897916).

**
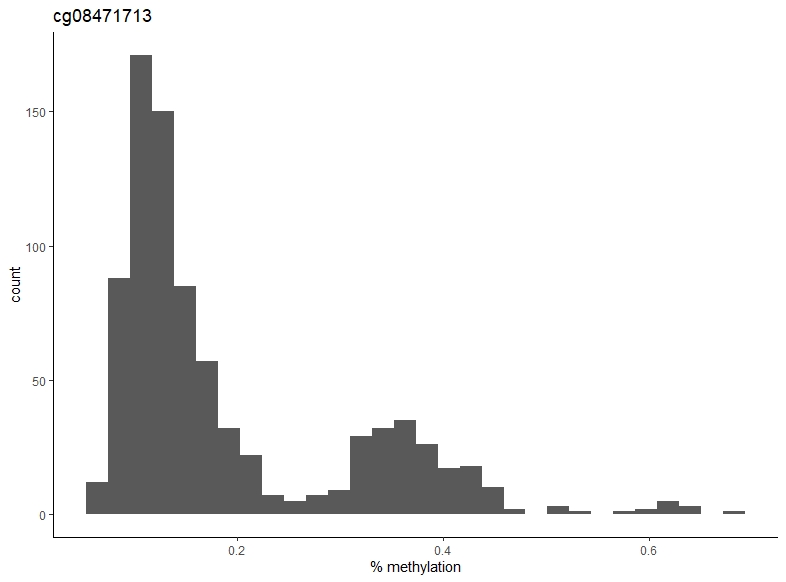
**

**Supplemental Figure 4.** Distribution of methylation levels at CpG probe cg08471713, Upstate KIDS


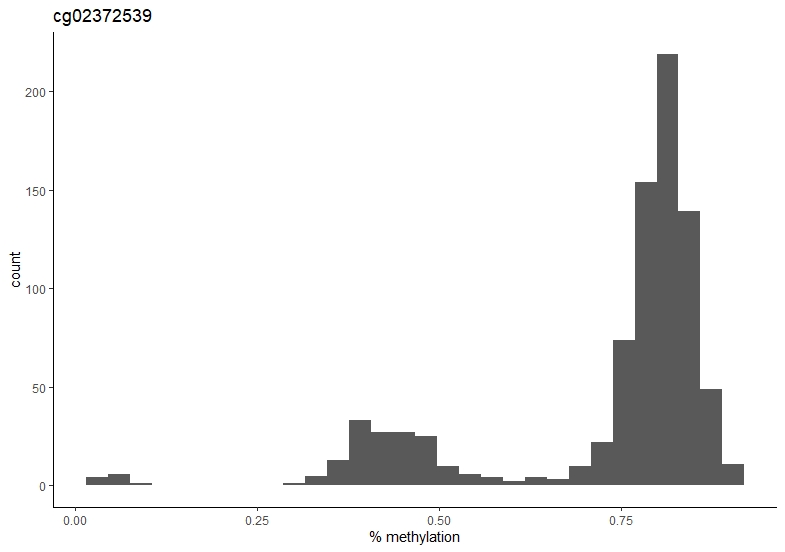


**Supplemental Figure 5.** Distribution of methylation levels at CpG probe cg02372539, Upstate KIDS

**Supplemental Figure 6.** Manhattan plot corresponding to Table 3 results of PCOS-hirsutism DMRs (fully adjusted model)


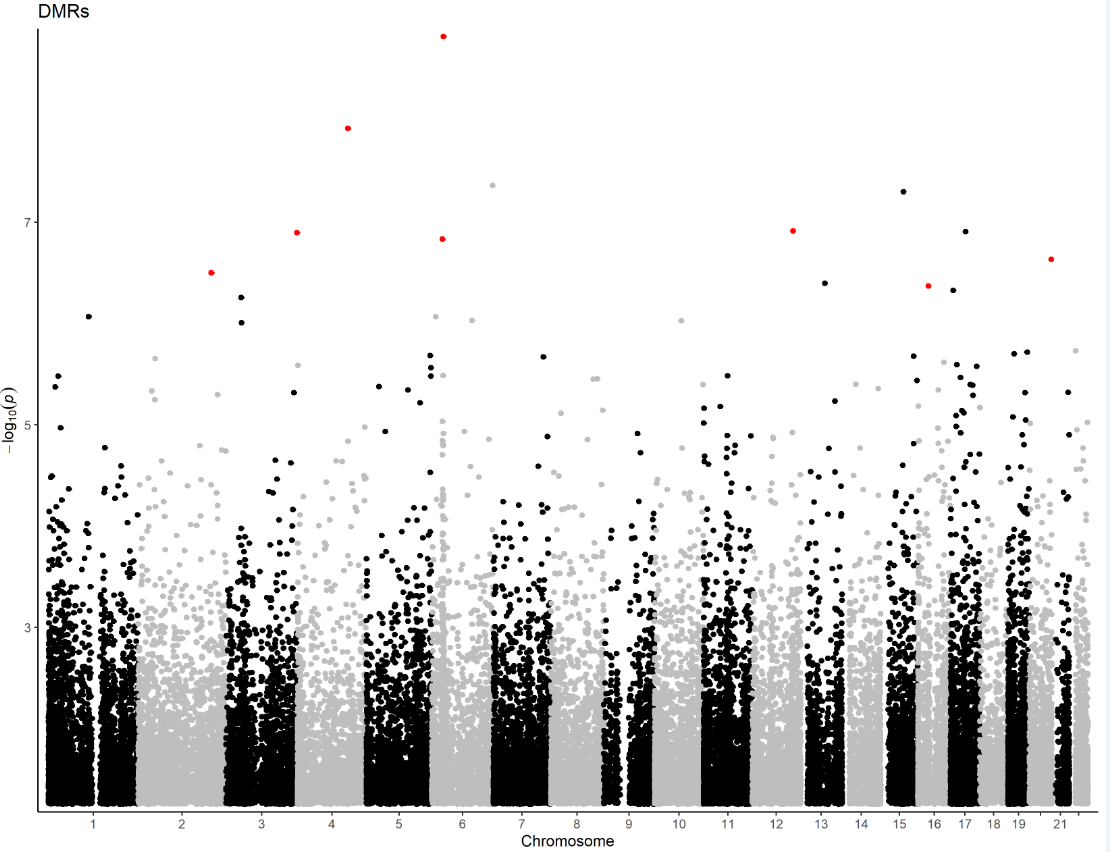


Legend: Colored dots denote the eight Bonferonni-significant DMRs identified in offspring newborn blood samples with exposure to maternal PCOS with hirsuitism compared to no PCOS.

**Supplemental Figure 7.** Volcano plot corresponding to Table 3 results of PCOS-hirsutism DMRs (fully adjusted model)


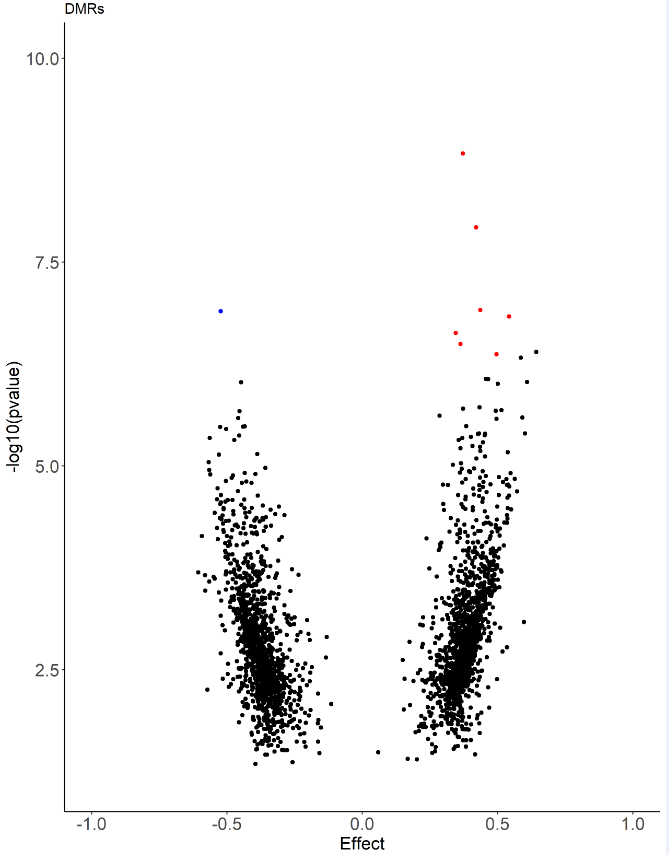


Legend: Colored dots denote the eight Bonferonni-significant DMRs identified in offspring newborn blood samples with exposure to maternal PCOS with hirsuitism compared to no PCOS (inputting the nominally significant results from the EWAS, p<0.05). The blue denotes less methylation with exposure and the red denotes more methylation with exposure.

**References**

1. Uhlén M, Fagerberg L, Hallström BM, Lindskog C, Oksvold P, Mardinoglu A, et al. Proteomics. Tissue-based map of the human proteome. Science. 2015;347(6220):1260419.

2. Yeung EH, Mendola P, Sundaram R, Zeng X, Guan W, Tsai MY, et al. Conception by fertility treatment and offspring deoxyribonucleic acid methylation. Fertil Steril. 2021;116(2):493-504.

3. Yeung EH, Guan W, Mumford SL, Silver RM, Zhang C, Tsai MY, et al. Measured maternal prepregnancy anthropometry and newborn DNA methylation. Epigenomics. 2019;11(2):187-98.
