# Supplementary material for: Examination of newborn DNA methylation among women with polycystic ovary syndrome/hirsutism
Source: Epigenetics. 2023 Nov 22;18(1):2282319. doi: 10.1080/15592294.2023.2282319 (PMC10732621; doi:10.1080/15592294.2023.2282319)
Supplement: KEPI_2023_0106R1 Response 10302023.docx [file KEPI_A_2282319_SM2009.docx]

KEPI-2023-0106R1

We thank the reviewers again for their time and insightful comments and the editors for giving us the opportunity to respond to this second round of review. Below are the point-by-point responses.

Reviewers' comments:

Please address the comments from reviewer 2 in a rebuttal. And, where possible, incorporate appropriate revisions into your manuscript. Please also follow the small revision suggested by reviewer 3. Let us know if you have any questions during the revision process.

**Reviewer 1:**

I believe the authors have satisfactorily addressed all the questions and made appropriate revisions to the manuscript.

**Response:** Thank you. We appreciate your time and efforts in reviewing our manuscript and for positive feedback on the revisions.
 **Reviewer 2:**

1) After review, the authors have answered most of the questions and the manuscript is now much clearer. The manuscript has great potential, but the data are poorly explored. Some plots are commonly used in array studies and give more information about the 850 points of DMRs, for example a Manhattan plot showing the distribution of DMRs across the genome, an interactome or gene ontology analysis of the DMRs with significant FDR, a heat map or volcano plot showing the DMRs (hiper and hipomethyalted). This is extremely important for this type of data.

**Response:** Thank you for the thorough review of our revisions. Below are the Manhattan and Volcano plots that have now been included as supplemental material in the revised manuscript.

Manhattan and Volcano plots corresponding to Table 2 findings:

Manhattan and Volcano plots corresponding to Table 3 findings:

*Note: The Volcano plot only include nominally significant (p<0.05) identified regions.*

We are uncertain of the reference to “850 points of DMRs”. However, we agree that if we observed many significant hits, the graphical displays suggested would be more compelling/useful in reviewing such large amounts of significant data points succinctly. However, each analysis had few, if any, hits which we felt were better captured in Table formats. Nonetheless, and in response to the reviewer, these Manhattan and Volcano plots are now added as **supplemental figures** as suggested. We also added the lambda values for the EWAS analyses to indicate lack of genomic inflation.

We ran a gene ontology analysis using the R package missmethyl in multiple ways; first including the EWAS specific CpGs where were FDR significant or at p<0.10 or p<0.20 (Table 2 for PCOS with hirsutism vs. not) and also using the list of significant DMRs identified (Table 3). There were no significant pathways identified whether relative to the promoter, gene body, or overall regardless of location. We now mention this additional analysis in our manuscript. An example of the missmethyl output on the DMRs is provided below for the reviewer/editorial board.

a) DMR analysis (p<0.05) – overall – only top 10 hits shown (all FDR p=1.00)

| Gene Ontology† | TERM | N genes in GO pathway | DE* | P-value for over-representation | FDR p-value | Significant gene in set |
| --- | --- | --- | --- | --- | --- | --- |
| BP | synapse assembly involved in innervation | 1 | 1 | 0.000282 | 1 | *FBXO45* |
| BP | regulation of cardiac endothelial to mesenchymal transition | 2 | 1 | 0.000725 | 1 | *GATA5* |
| BP | positive regulation of cardiac endothelial to mesenchymal transition | 2 | 1 | 0.000725 | 1 | *GATA5* |
| BP | cardiac endothelial to mesenchymal transition | 3 | 1 | 0.001532 | 1 | *GATA5* |
| CC | NatA complex | 5 | 1 | 0.002557 | 1 | *NAA15* |
| CC | rod photoreceptor outer segment | 6 | 1 | 0.003064 | 1 | *TMEM237* |
| BP | Notch signaling pathway involved in heart induction | 4 | 1 | 0.003141 | 1 | *GATA5* |
| BP | regulation of Notch signaling pathway involved in heart induction | 4 | 1 | 0.003141 | 1 | *GATA5* |
| BP | positive regulation of Notch signaling pathway involved in heart induction | 4 | 1 | 0.003141 | 1 | *GATA5* |
| BP | endocardial cushion fusion | 5 | 1 | 0.003391 | 1 | *GATA5* |

† BP, biological process; CC, cellular component (Gene Ontology terminology)

*DE = # of genes differentially methylated

2) In addition, all genomic data must be available in the Gene Expression Omnibus (GEO). The participant's data are not required, but the array information for both Upstate KIDS and EAGeR must be included before the manuscript can be accepted. Authors can submit to receive the accession number and schedule the release of the data after acceptance of the manuscript. Please provide the GEO accession number for this study.

**Response:** The NIH GDS policy came into effect January 2015. EAGeR completed all data and specimen collection by 2011. Upstate KIDS originally collected the blood spots for which DNA was derived also prior to this date. Hence, both studies did not include language in the original consent forms that have been approved for deposits into public databases by an IRB. We agree with data sharing to the maximal degree allowed. We have been (and will continue to be) very collaborative with any institutions which wish to access the data through Data Use Agreements (as one IRB deemed that the consent language only allows sharing under the stipulation that individuals are working with NICHD) and have already specified in the manuscript that: Data that support the findings of this study are available on request from the corresponding author [EY]. Data are not on a public database due to privacy restrictions (i.e., releasing information that could compromise participant privacy/consent).

Please understand that using blood spots collected from the Newborn Screening Program in research has been hugely contested in multiple states in the US, resulting in millions of them being destroyed (e.g., [MI and TX](https://apnews.com/article/michigan-newborn-blood-spots-constitution-d3db6a3d09ae65d7c7054a0f483c6833#:~:text=In%202022%2C%20at%20a%20separate,department%20has%20defended%20the%20program.)). Accordingly, the IRB is being reasonably strict as to who can legally access data derived from the materials even when it’s not attached to identifiers and have no obvious privacy risk.

3) Regarding question 4, I disagree with the authors. The 850k methylation probes provide this information and it is possible to score these CpG elements and the lack of numbers is unusual in a genome-wide study. I still suggest that the authors include figures and a table in the supplementary information.

**Response:** We now include the plots suggested above. We remain unclear on the interpretability and validity of the summary statistics being requested by the reviewer. Even though there are a large number of CpG sites included on the 850K array, the probes are specifically designed by Illumina to provide comprehensive coverage on the gene coding and regulatory regions rather than unbiased coverage on the entire genome (Moran S, Arribas C, Esteller M. Epigenomics. 2016 Mar;8(3):389-99. *Validation of a DNA methylation microarray for 850,000 CpG sites of the human genome enriched in enhancer sequences.* Pidsley et al. Genome Biol. 2016 Oct 7;17(1):208. *Critical evaluation of the Illumina MethylationEPIC BeadChip microarray for whole-genome DNA methylation profiling.*). It is different from a GWAS array which is designed by the tagging SNP approach to cover most of common SNPs on the genome. We are therefore hesitant to estimate the whole-genome methylation level using these array data until we can find a reference to confirm the validity of such approach (which we failed to locate through PubMed/google scholar search).

4) The participants in the EAGeR study should be clarified. What are the clinical features of infertility or subfertility? Detailed inclusion and exclusion criteria should be provided. Although the authors have provided a flowchart of the study, I could not understand the information, it is very confusing. Out of 4889 mothers, 3107 were included because they agreed to participate. No other exclusions were made? What does genetic consent mean? 855 unrelated newborns were singletons? the final number for upstake children. 849 newborns were born to 102 mothers (69+33)? Please give the information about the PCOS newborns with and without hirsutism.

**Response:** For EAGeR we have added the following text to page 5-6 of the methods section (new text bolded):

“**Eligibility for the trial included women aged 18 to 40 who 1) had 1-2 prior pregnancy losses, 2) ≤ 1 prior live birth, 3) ≤ 1 elective termination or ectopic pregnancy, 4) regular menstrual cycles for 21-42 days in the prior year.** Exclusion criteria included any prior **history** of infertility or sub-fertility including conditions like PCOS **or currently undergoing or planning use of medical fertility treatments**.”

For the Upstate KIDS study, it is correct that out of 4889 mothers, 3017 provided consent for retrieval of their newborn’s dried blood spots (DBS) in 2009-11 for the purpose of measuring proteins and environmental chemicals. Thus, we sought additional consent to assess DNA from the DBS samples already collected back in 2009-11 because the original consents did not give permission to conduct genetic analyses. We received permission for 1071 children. This number included twins from the same family and was derived prior to data cleaning steps. Hence, after removing 47 infants with insufficient DNA, 1024 samples were run from 869 families, excluding 155 related twins, 8 with sex mismatch and 9 with missing rate >3% in analysis. The resulting 855 unrelated newborns include all singletons and a randomly selected twin from a pair with methylation data. All details have been previously published. The 849 were born to 69 mothers with PCOS without hirsutism, 33 with PCOS and hirsutism, and 747 without PCOS. To help clarify this issue, we revised the order of information on page 5 as follows to better supplement the figure (bolded text corresponds to modified text):

“**Parents provided written informed consent for the retrieval of remaining newborn dried blood spots (DBS) from New York State’s Newborn Screening program when the infants were 8 months old (2009-2011).** **Samples were retrieved for the measurement of immunoglobulins and eluted spots were archived.** **Parents (of 1071 children) provided additional genetic analysis consent in 2016-2017 to measure DNA methylation from the DBS (24).** For this analysis, a subset of 855 singletons and twins (one randomly selected twin from each pair) with DNA methylation (DNAm) measured were included, as previously described (23). Follow-up of cohort participants continued until 2019. The New York State Department of Health and the University at Albany (State University of New York) institutional review boards (IRB) approved the study.”

5) Table 4 is confusing and not very informative. The quartile 4 is very broad, a woman with a testosterone level of 73.99 is likely to have hyperandrogenism while 26.35 is not. Did the authors analyse DNA methylation with this stratification in mind?

**Response**: Table 4 provides summary descriptive statistics to orient the reader to who the participants were in the EAGeR sample and how covariates varied in relation to testosterone levels. We understand the author’s concern that these testosterone quartiles are not related to clinical cutoffs, its limitation for diagnostic use we have already acknowledged (p17).

Were the spots in the Upstate KIDS study blots from the heel prick test and is it not possible to compare DNA methylation between the children with PCOS and hirsutism and EAGeR with high testosterone levels?

**Response**: That is correct, the dried blood spots in the Upstate KIDS study were from the heel prick test conducted as part of the Newborn Screening Program. We did not compare any dried blood DNAm from the Upstate KIDS participants with the cord blood DNAm from any EAGeR participant as the array is known for batch effects and the samples from each study were not run on the same plates (and not even in the same year). We now mention that the microarrays to measure DNA methylation in the two studies were not conducted concurrently and data could not be combined (p17).

In addition, the DNAm analysis in Table 5 should be stratified by testosterone level.

**Response**: Supplemental Table 3 provides the analyses by testosterone quartiles as a follow-up to Table 5 which shows the results of continuous testosterone (log-transformed) with DNA methylation. Given the limited sample size in EAGeR and high burden of multiple testing, we have limited statistical power in stratified analysis.

6) The methods section should read "Participants, material and methods" as this study involves human material.

**Response:** We have revised the section heading.

7) Please provide the Institutional Review Board (IRB) approval number for this current work and the registration number for the EAGeR clinical trial.

**Response:** These numbers have been added.

8) Please provide the description of EAGeR in the subtitle of the methods section

**Response:** We have added the following to page 5 (new text bolded):

“In an exploratory analysis to evaluate concentrations of hormones, we used data from the Effects of Aspirin in Gestation and Reproduction (EAGeR) trial (2007-2011)**. EAGeR** was a multicenter, double-blind clinical trial that randomized 1,228 women to low-dose aspirin and folic acid vs placebo and folic acid prior to conception (25, 26). **The primary objective of the trial was to examine whether preconception-initiated low dose aspirin improved live birth among women with a history of pregnancy loss. Data collected as part of this unique preconception trial have been leveraged into other epidemiologic investigations of the reproductive process**.”


**Reviewer 4:**

I am satisfied the points I raised have now been addressed.
Just one minor point - there is some omitted text in the revised Discussion - "we cannot" ... is missing from the middle of the sentence below;

"While preconception testosterone is more typical of T concentrations than those measured throughout pregnancy, account for diurnal rhythms due to availability of a single measure which was not meant for diagnostic purposes."

**Response:** Thank you your additional review of our manuscript. We added the omitted text back into the Discussion.
